# Bridging Health Disparity Gaps in Alzheimer’s Disease among Marginalized Populations: Clinical Proteomics as a Case Study

**DOI:** 10.1021/acsbiomedchemau.5c00074

**Published:** 2025-07-08

**Authors:** Henry A. Adeola, Renã A. S. Robinson

**Affiliations:** † Department of Chemistry, 5718 Vanderbilt University, Nashville, Tennessee 37235, United States; ‡ Department of Oral and Maxillofacial Pathology, Faculty of Dentistry, University of the Western Cape and Tygerberg Hospital, Francie Van Zijl Dr, Cape Town 7505, South Africa

**Keywords:** Alzheimer’s disease, ADRD, clinical proteomics, marginalized populations, dementia, health disparities

## Abstract

Alzheimer’s disease (AD) and AD-related dementias (ADRD) represent a significant health challenge, with a growing impact on marginalized populations who often experience inequities in overall healthcare access and outcomes. Many factors contribute to these inequalities and can impact the benefits of broad appreciation of new technologies in AD/ADRD to these populations. For example, clinical proteomics offers a promising avenue for early and timely detection of disease and elucidation of the mechanisms of AD/ADRD. Unfortunately, gaps exist in the access and application of proteomic innovations for the health of marginalized communities. This editorial (1) highlights systemic barriers and explores the underlying factors that contribute to these inequities, (2) examines health disparities in the implementation of clinical proteomics tools for the management of AD/ADRD among marginalized populations, and (3) offers opportunities for advancing clinical proteomics in AD/ADRD. Implementation by basic and clinical researchers will lead to a more effective and inclusive approach to combatting AD/ADRD disparities.

## Graphical abstract

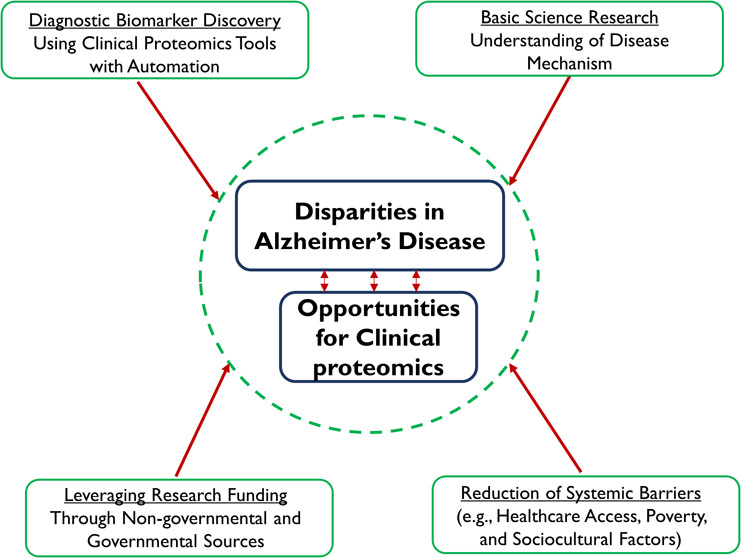

## 1 Introduction

Alzheimer’s disease (AD) and AD-related dementias (ADRD) are a public health challenge that affect millions of people globally.^1^ AD is the most common cause of dementia in the elderly population.^2,3^ Greater than a trillion US dollars are required for the management of AD worldwide.^3^ AD/ADRD is the fourth leading cause of disability-adjusted life years (DALYs) lost in persons ≥75 years of age.^4^ In 2023, the World Health Organization (WHO) estimated that by 2050, ∼139 million people will suffer from dementia, of which greater than 60% reside in low- and middle-income countries (LMICs).^5^ As the global population ages, the prevalence of AD/ADRD is expected to rise significantly.^5^ This will have profound fiscal and infrastructural implications for healthcare systems and further widen the healthcare access gap between the affluent and the most vulnerable and underserved communities.

Considerable progress has been made in improving our understanding of the molecular pathophysiology of AD/ADRD and has led to substantial investments in therapeutics and diagnostics based on markers such as amyloid-β (Aβ) and the major microtubule-associated protein *tau*.^6^ However, effective treatments and diagnostic avenues remain elusive, and there are only two Food and Drug Administration (FDA)-approved therapies (lecanemab and donanemab) for AD that may slow disease progression.^7^ One FDA-approved AD therapy (aducanumab) was recently discontinued.^8^ Disease diagnosis has been better facilitated by combining probes such as positron emission tomography (PET) and magnetic resonance imagining (MRI) scans^9^ with cerebrospinal fluid (CSF), blood, and other biospecimen-based markers^10,11^ and traditional clinical approaches.

Clinical proteomics (CP), the large-scale study of the full complement of proteins in biological systems,^12^ holds great promise for unraveling the complex molecular mechanisms underlying AD/ADRD and for accelerating diagnostic and therapeutic avenues for ADRD.^13^ However, a broad-based translation of proteomic findings into clinical practice has been hindered by various modifiable factors such as disparities in research funding, as well as technical and logistic limitations in access to technology, cost, infrastructure, human resources, statistical modeling, data harmonization/integration, data deposition, and underrepresentation of marginalized populations.^14–16^ Minority populations, including but not limited to ethnic and racial minorities, indigenous populations, as well as socioeconomically disadvantaged groups, bear a disproportionate burden of AD/ADRD prevalence and related risk factors.^17–20^ Unfortunately, they are often underrepresented in clinical research,^21^ leading to a lack of diversity in proteomic data sets and limited generalizability of findings. CP research has also predominantly focused on populations in high-income countries, neglecting the unique challenges faced by marginalized communities in LMICs.^22^ Given the expected increase of older adults in marginalized communities by 2050,^23^ there is an urgency to ensure AD/ADRD research is advancing. This editorial aims to elucidate health disparities in the utilization of CP for AD/ADRD among marginalized populations, identify contributing factors, and propose strategies to address these disparities.

## 2 Risk Factors and Determinants of AD/ADRD among Marginalized Populations

Most AD cases are sporadic, although between 5 and 10% of cases have familial Mendelian-type genetic inheritance patterns.^24,25^ Beyond nonmodifiable risk factors such as genetics, age, sex, and race, some other modifiable risk factors include chronic systemic conditions such as hypertension and diabetes, as well as other social risk factors such as depression, physical inactivity, poor sleep quality, low education level, and low level of social interaction.^26^ A major genetic risk factor for sporadic or late-onset AD (LOAD) is the apolipoprotein E (*APOE*) ε4 allele.^27^ LOAD typically occurs after the age of 65 years and is characterized by progressive impairment of higher intellectual function and memory as age increases, leading to loss of multiple cognitive functions.^27,28^ Conversely, the nonsporadic familial mendelian-type AD leads to early-onset AD (EOAD). The most common occurring genetic risk loci for the EOAD include chromosomes 21, 14, and 1, which harbor amyloid-β (Aβ) precursor protein (APP), presenilin 1 (PSEN1), and presenilin 2 (PSEN2), respectively.^29^ Consequently, adult patients with Down syndrome (trisomy 21) who survive beyond the age of 45 years tend to develop neurocognitive decline and clinicopathological features of AD.^30–32^

The risk of developing AD/ADRD also differs by ethnicity, race, and gender.^19,20,33–35^ Caribbean-Latino and African American adults were observed to have a higher burden of AD/ADRD as compared to non-Hispanic White adults.^34,36^ Women are twice as likely to develop AD/ADRD as compared with men, particularly after the age of 60 years.^37,38^ Plausible explanations for this gender disparity include differences in *APOE* ε4 allele risk, neurodegenerative shortening of telomeres, psychosocial comorbidities (insomnia, higher depression risk, and low educational levels), as well as pregnancy-related disorders (preeclampsia and gestational hypertension) that are capable of contributing to disparities in cognitive reserve. Genetic risk factors such as mutations in Adenosine Triphosphate-Binding Cassette, Subfamily A, Member 7 (*ABCA7*), *APOE*, Bridging Integrator 1 *(BIN1)*, Triggering receptor expressed on myeloid cells 2 *(TREM2)*, WW domain-containing oxidoreductase (*WWOX*), CD2 associated protein (*CD2AP*), and FERM Domain Containing Kindlin-2 (*FERMT2*), have been implicated in African American adults with AD.^39^

ABCA7 is a lipid-transporter that, when mutated, can disrupt key biological processes such as lipid homeostasis and phagocytosis.^40^ In fact, the *ABCA7* gene has been identified as possessing stronger associations with risk of AD in people of African ancestry as compared with those of European ancestry.^40^ Mutation of the APOE ε4 allele affects the aggregation of amyloid plaques as well as the development of neurofibrillary tangles.^41^ BIN1 mutation is the second most important AD risk gene that has been identified to affect calcium homeostasis in human brain glutamatergic neurons at the later stages of AD pathogenesis.^42^ Mutation in the TREM2 gene, which is a receptor involved in pattern recognition and is well-expressed in microglia, is also known as an important risk factor for AD pathology.^43^ By binding to Tau via the c-terminal Short-chain dehydrogenase/reductase domain, WWOX blocks AD progression by interacting with key Tau phosphorylating enzymes such as GSK-3β, ERK, and JNK.^44^ Mutation of this gene can lead to AD pathogenesis. CD2AP is a scaffolding molecule that regulates cytoskeletal molecules and signal transduction, and its mutation has been implicated in AD pathogenesis.^45^ Mutation in the FERMT2 (also known as Kindlin-2), a protein involved in the regulation of synaptic plasticity and axonal growth, has been reported to directly modulate the metabolism of APP and thus contribute to the development of AD.^46^

Genetics, aging, sex, ethnicity, and race are nonmodifiable risk factors; thus, modifiable lifestyle risk factors can be leveraged to mitigate the development and progression of AD/ADRD among high-risk populations.^47^ Nongenetic risk factors such as multidimensional poverty, psychosocial stress, and chronic low-grade infection may play roles in the epidemiology of AD among the elderly African population.^34^ However, inequities in AD risk are poorly researched in multiethnic AD/ADRD cohorts.^34^ Pervasive disparities in access to other structural and social determinants of health, such as healthcare, employment opportunities, living environments, quality of education, and discrimination and oppression (racism and classism), have been tied to AD/ADRD burden among African American communities.^20,48–50^ Rubin *et al*. found that well-established genetic risk factors of AD/ADRD were poorly researched among U.S. minority populations.^51^ Similarly, dementia risk factors based on cardiovascular conditions, physiological variables, and lifestyle factors were identified as most prevalent among African American/Black participants from the National Alzheimer’s Coordinating Center (NACC) database.^52^

## 3 Current Advances in the Diagnosis and Treatment of AD/ADRD

Clinical proteomics entails the study of proteins in various clinical samples (e.g., blood plasma/serum, CSF, saliva, etc.) on a global scale. Despite the promising potential of using human brain tissues as a target organ for AD biomarker discovery, a key drawback is the impediment in isolating and analyzing specific subpopulations of cells, e.g., glia or neuronal cells, from different regions of the brain.^53^ Additionally, brain tissue is acquired post-mortem.^54^ Variability within or between regions of post-mortem brain tissues also can lead to interpretation difficulties.^55^

Clinical proteomics has traditionally used targeted approaches to measure proteins (e.g., Aβ, tau, ApoE, BACE, NfL, TDP-43), which limits the discovery of other novel and potential biomarkers of AD/ADRD.^6,56,57^ Conventional protein approaches such as Western blotting and enzyme-linked immunosorbent assay (ELISA) have evolved to high-throughput array-based or mass spectrometry (MS)-based proteomics methods,^58^ due to the requirement for scale, cost, and availability. In 2020, the Alzheimer’s Drug Discovery Foundation Diagnostics Accelerator supported a biomarker research effort that led to the discovery of the first blood test for the detection of brain amyloid plaque by the C2N Diagnostics company.^59^ Subsequently, in 2024, a large clinical study evaluated the diagnostic accuracy of C2N’s PrecivityAD2 blood test for AD in primary and specialized care settings and documented strong performance robustness in both care settings.^60^ This MS-based blood test analyzes plasma samples to evaluate the ratio of phosphorylated tau 217 (p-tau217) to non–p-tau217. An accuracy, sensitivity, and specificity of over 90% were found for the C2N’s PrecivityAD2 blood test when compared with amyloid PET and CSF analyses in over 1,200 patients.^60,61^ Even though there are currently no FDA-approved blood tests for AD/ADRD, there has been a rapid evolution of clinical proteomics instrumentation (Figure 1A) as well as novel potential biomarkers, which are in the pipeline for approval (Figure 1B).

**Figure 1:**
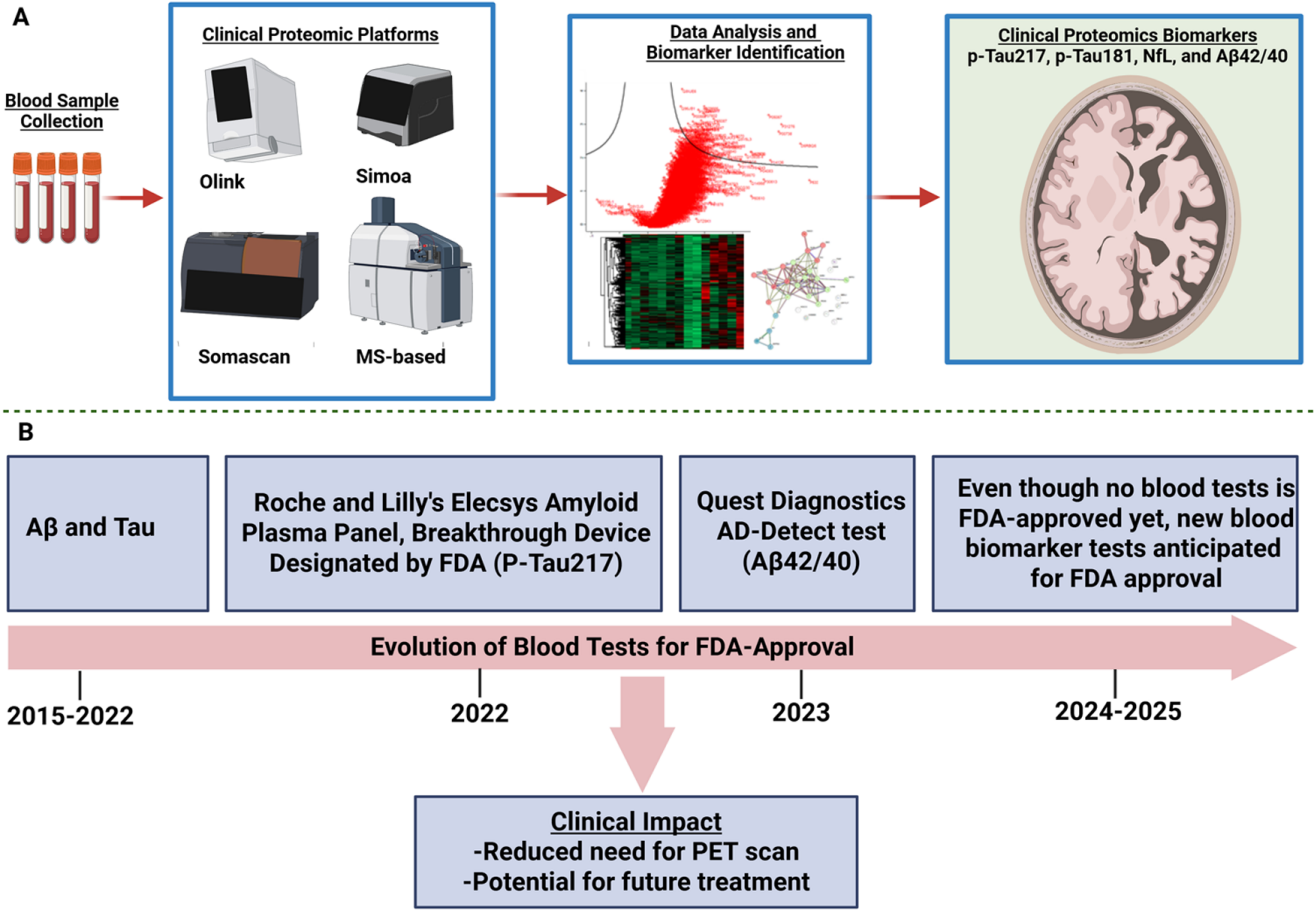
Emerging instruments and analytical approaches for the implementation of clinical proteomics biomarker discovery from blood samples (A) and a timeline highlighting developments from clinical proteomics on blood test development for ADRD (B) (Figure 1A partially generated in Biorender).

On the policy level, the National Alzheimer’s Project Act (NAPA) subcommittee has called on various stakeholders, such as the Center for Diseases Control (CDC), the National Institute of Health (NIH), the Indian Health Service, the Veterans Administration, the Health Resources and Services Administration, the Centers for Medicare & Medicaid Services, Administration for Community Living and the Aging Services Network, and others, to support a comprehensive, equitable, and inclusive intervention for the assessment, and reduction of dementia risk in a manner that fosters proportional representation of minority populations.^62^ Various FDA-approved drugs, such as aducanumab (Aduhelm), lecanemab (Leqembi) and donanemab (Kisunla), Memantine (Namenda), Donepezil (Aricept), Galantamine (Razadyne), Rivastigmine (Exelon), and others, have emerged over time for the management of AD/ADRD, albeit some have been discontinued, and most are disease-modifying and are mostly useful in early and middle disease stages.^63–70^ There is still no cure for AD or ADRD.

## 4 Barriers and Challenges to Application of Clinical Proteomics for AD/ADRD

Several barriers contribute to disparities in the application of CP for research and the clinical management of AD among minority populations. A few broad-based examples will be discussed hereafter. First, healthcare disparities deserve to be viewed not only from an individual discriminatory perspective but rather from a contextual lens of systematized racial inequalities in societal health institutions.^71–73^ Second, the root cause of disparities are nuanced and multidimensional, and as such, involves a complex interplay between the geographic location of healthcare facilities, educational level, insurance coverage, socioeconomic status, discriminatory patient (and minority provider) practice in the managed care system, unconscious bias, and other factors.^74^ Third, egalitarian principles that are the core value of the American society need to be actively engaged to eradicate the healthcare gap that racial inequalities breed among minoritized Americans. Utilization of emerging CP tools for AD/ADRD, collaborations, global partnerships, and knowledge-sharing is important for the development and sustenance of proteomics research in resource-scarce regions and among minoritized populations. The application of CP for the diagnosis and treatment monitoring of AD/ADRD and other diseases is currently lacking in many LMICs due to known barriers such as high costs, lack of infrastructure, poor resource allocation, language barriers, as well as unfavorable government regulations.^75–77^

### 4.1 Healthcare Access and Utilization Disparities

Various institutional proteomics platforms and core facilities have been established across the United States, providing precision clinical care for risk stratification, diagnosis, and targeted therapies.^78,79^ However, despite the promise of CP, marginalized communities often face barriers to accessing healthcare services, including diagnostic tests and specialized treatments,^80^ and in research participation. Limited participation in CP research hinders the development of culturally sensitive interventions.^80–82^ Currently, African American populations experience long-term disparities in healthcare access as well as health outcomes,^74^ which include higher uninsured rates and higher likelihood to be deprived of adequate healthcare due to (personally unaffordable) costs, as well as having poor health status.^74,83^ Over the past decade, life expectancy in the African American population has been 3–6 years shorter compared to the non-Hispanic White population.^84–86^ Even if CP tools were provided in major healthcare facilities, the aforementioned disparities would limit the access of marginalized populations despite goals to achieve equitable healthcare for all.^74^ Beyond differences in access and utilization of CP for marginalized populations, socioeconomic factors, such as inadequate education, poverty, and the social stigma surrounding dementia, can act as a deterrent to individuals from seeking medical help or participating in research studies.

### 4.2 Socioeconomic Factors

Limited resources for research infrastructure and personnel in regions where marginalized populations seek healthcare constrain the capacity for CP research. CP has the potential to ameliorate bottlenecks in the clinical diagnosis of disease. However, when compared to genome sequencing costs, which stood at ∼$100 per sample in 2022,^87^ proteomics analysis costs have been found to be ∼$375 per sample,^87,88^ which reduces the accessibility of CP. Recent National Institute on Aging (NIA) research indicated that racial and ethnic disparities were observed in dementia care costs.^89^ Between 2000 and 2016, the adjusted mean total Medicare expenditures for non-Hispanic Black and Hispanic populations (≥65 years of age) were significantly higher in comparison to those of non-Hispanic White populations ($165,730 vs $160,442 vs $136,326, respectively).^89^ Even though these disparities were observed across the manifold phases of dementia care, it was emphasized that some of the differences in care utilization were probably a result of cultural and patient factors, caregiver preferences, disparities in life-end follow-up visit frequency, dementia care coordination, and access communication for different care options. Despite paying higher healthcare costs and having a disproportionately higher risk of developing AD/ADRD, marginalized populations are more likely to receive subpar dementia care.

### 4.3 Linguistic and Cultural Diversity

The delivery of high-quality healthcare can be impeded by language barriers, and this can have a negative impact on patient safety, healthcare quality, and the sense of fulfillment of patients and healthcare providers.^90^ In the United States, cross-cultural and racial health disparities are well documented, and health indicator disparities have been found between the general populace and minoritized groups such as Native Americans, Latino Americans, and African Americans.^91,92^ Language barriers, cultural beliefs, and mistrust of research institutions can negatively impact the willingness of minority populations to participate in proteomic studies.^93^ It is customary in many CP studies to exclude papers that are not written in English and other major languages from systematic reviews and meta-analyses,^94,95^ thus limiting the amount of research that can be conducted among marginalized populations. Language barriers in healthcare systems result in miscommunication between patients and medical professionals. Hiring language interpreters, where needed, indirectly adds to the cost and time spent in treatment visits.^90^ The U.S. Department of Health and Human Services (DHHS) Office for Civil Rights deems insufficient interpretation during healthcare consultations as a form of discrimination, a view upheld by the DHHS from the Civil Rights Act of 1964.^96^ Cultural competency and community engagement are essential for building trust and facilitating meaningful collaborations. In spite of the federal government mandates to ensure inclusion of women and minoritized populations in federally funded research, Black populations are still highly unlikely to participate in research studies compared to White populations.^93^ Due to mistrust, lower rates of Black population participation have been reported in many disease studies,^97–100^ including AD/ADRD.^101^

### 4.4 Proteomic Research Data Bias and Generalizability

Despite the clinical utilities of emerging proteomic biomarkers of AD/ADRD, overlooking racial differences in the expression profile of these biomarkers could lower healthcare quality.^19^ The lack of diversity and inclusion of minority groups in proteomic data sets systematically dampens the identification of novel biomarkers and therapeutic targets that are relevant to minority populations.^102^ Without adequate representation, research findings may not be applicable or effective in addressing the needs of these communities.^19,102,103^ A significant number of reported AD studies in the last decade lack explicit information on the inclusion of African American adults or other minoritized groups in their study design (Table 1). Of these 34 studies, 29 (85.3%) do not include clear information on inclusion of marginalized populations, while only five (14.7%) contained detailed information on inclusion of African American and Latino American groups. In addition, a majority of the studies were carried out using post-mortem brain tissues (29.4%) and invasive samples such as CSF (50%). Only ∼20% included plasma as the study sample.

**Table 1:** Summary of 34 Key Studies over the Past Decade That Used Clinical Proteomics AD/ADRD with Demographic Information

clinical proteomics studies for AD/ADRD	clinical proteomics approach	key proteins identified	population demographics	type of tissues samples used	year published
Seifar *et al.* ^104^	MS	9899	1385^a^ ^,^ ^b^ ^,^ ^c^	brain	2024
Nilsson *et al.* ^105^	MS and immunoprecipitation	SNAP-25, 14-3-3 zeta/delta, β-synuclein, and neurogranin	958 participants from the Swedish BioFINDER-2 study, Skåne University Hospital, Lund, Sweden^d^	CSF	2024
Haque et al.^106^	MS	48	706 individuals from ADNI^d^	CSF	2023
Johnson et al.^107^	MS	33	475 samples from DIAN at Washington University^d^	CSF	2023
Montoliu-Gaya et al.^108^	IP/MS	p-tau181, p-tau199, p-tau202, p-tau205, p-tau217, p-tau231, tau195–205 and tau212–221	214 participants from the Paris Lariboisière and Translational Biomarkers of Aging & Dementia cohorts^d^	plasma	2023
Modeste et al.^103^	MS	402	203^a^ ^,^ ^c^	CSF	2023
Weiner *et al.* ^109^	MS and CLEIA	SCRN1 and tau	244 patients from University of California San Diego Shiley–Marcos Alzheimer’s Disease Research Center^d^	CSF	2023
Dammer et al.^110^	MS, Olink, and SomaLogic SomaScan	>9500	154 samples from the Emory ADRC^d^	brain, CSF, and plasma	2022
Gobom et al.^111^	MS and Simoa	pT181, pS199, pS202, pT205, pT217, pT231, and pS396	38 samples from Sahlgrenska University Hospital, Mölndal, Sweden^d^	CSF	2022
Johnson et al.^112^	MS	8600	516 from ROSMAP^d^	brain	2022
Dey et al.^113^	MS	>3000	16 samples from Banner Sun Health Research Institute^d^	CSF	2022
Carlyle et al.^114^	Olink	414	54 subjects from Massachusetts ADRC cohort^d^	plasma	2022
Kirmess et al.^115^	MS	APOE2/2, APOE2/3, APOE2/4, APOE3/3, APOE3/4, APOE4/4, Aβ40 and Aβ42	5 pooled samples for C2N Diagnostics commercial PrecivityAD Test^d^	plasma	2021
Bai et al.^116^	Meta-analysis	2698	7 deep data sets^d^	brain, CSF, serum	2021
Khan et al.^102^	MS	25	113^a^ ^,^ ^c^	plasma	2021
Florentinus-Mefailoski et al.^117^	MS	50	24 samples from Vrije Universiteit Amsterdam^d^	plasma	2021
Tijms et al.^118^	MS and ELISA	705	552 participants from EMIF-AD MBD and the ADNI^d^	CSF	2020
Bader et al.^119^	MS	867	197 from Sweden & Magdeburg/Kiel cohorts^d^	CSF	2020
Higginbotham et al.^120^	MS	3691	40 samples from the Emory ADRC^d^	CSF	2020
Ping et al.^121^	MS	8415	27 samples from Emory ADRC^d^	brain	2020
Stepler et al.^122^	MS	568	55^a^ ^,^ ^c^	brain	2020
Barthélemy et al.^123^	MS and Immunoassay (pT181 and t-tau)	tau	474 by the DIAN^d^	CSF	2020
Zhou et al.^124^	MS	SMOC1, YWHAZ, ALDOA and MAP1B	88 from the Emory ADRC^d^	CSF	2020
Shi et al.^125^	SOMAscan	44	881 from EMIF-AD MBD study^d^	plasma	2019
Sathe et al.^126^	MS	139	10 samples from BIOCARD study^d^	CSF	2019
Dey et al.^127^	MS	30	11 collected from Banner Sun Health Research Institute^d^	serum	2019
Morris et al.^128^	ELISA	Aβ42, p-tau181, t-tau	1255^a^ ^,^ ^c^	CSF	2019
Whelan et al.^129^	Olink ProSeek immunoassay	270	1022 samples from the Swedish BioFINDER study^d^	CSF and plasma	2019
Ovod et al.^130^	IP/MS	Aβ38, Aβ40, and Aβ42	41 participants from Washington University ADRC^d^	plasma	2018
Johnson et al.^131^	MS	350	47 samples from Emory ADRC^d^	brain	2018
Abreha et al.^132^	MS	1682	10 samples from the Emory ADRC^d^	brain	2018
Hales et al.^133^	MS	2771	35 samples from the BLSA^d^	brain	2017
Dammer et al.^134^	MS	142	16 samples from the Emory ADRC^d^	brain	2015
Sattlecker et al.^135^	SOMAscan	13	691 samples from AddNeuroMed biomarker study^d^	plasma	2014

a African Americans.

b Hispanic/Latino Americans.

c Non-Hispanic White.

d Unknown population breakdown; ADNI - Alzheimer’s Disease Neuroimaging Initiative; ADRC - Alzheimer’s Disease Research Center; BIOCARD - Biomarkers for Older Controls at Risk for Dementia; BLSA - Baltimore Longitudinal Study of Aging; CLEIA - Chemiluminescent enzyme immunoassay; DIAN - Dominantly Inherited Alzheimer Network; ELISA -Enzyme-linked immunosorbent assay; EMIF-AD MBD - European Medical Information Framework for Alzheimer’s Disease Multimodal Biomarker Discovery; IP/MS - immunoprecipitation mass spectrometry; MS - Mass Spectrometry; ROSMAP - Religious Orders Study and Memory and Aging Project.

The identification of AD/ADRD-related protein biomarkers is crucial for early detection, risk stratification, and precision treatment monitoring among minoritized populations. Statistical methods that can evaluate the effects of multiple rather than single AD/ADRD predictors are more informative when considering designing disparities research for dementia.^21^ Poor access to innovative therapies in underserved communities can severely impact patient outcomes, thus exacerbating the dismal burden of AD/ADRD among ethnic minorities.

## 5 Potential Opportunities for Improvement of AD/ADRD Care among Marginalized Populations

The causative factors and the consequences of AD/ADRD health inequities in the United States are nuanced.^49^ In order to address disparities in the usage of CP, a multifaceted approach that involves stakeholders at various levels is urgently required to reduce (or eliminate) the detrimental effects of AD/ADRD inequalities.^48^ Innovative solutions should be developed to bridge these disparities to achieve the desired outcomes for AD/ADRD among marginalized groups (Figure 2). Hence, a few suggestions to potentially realize this goal are presented in the following section.

**Figure 2:**
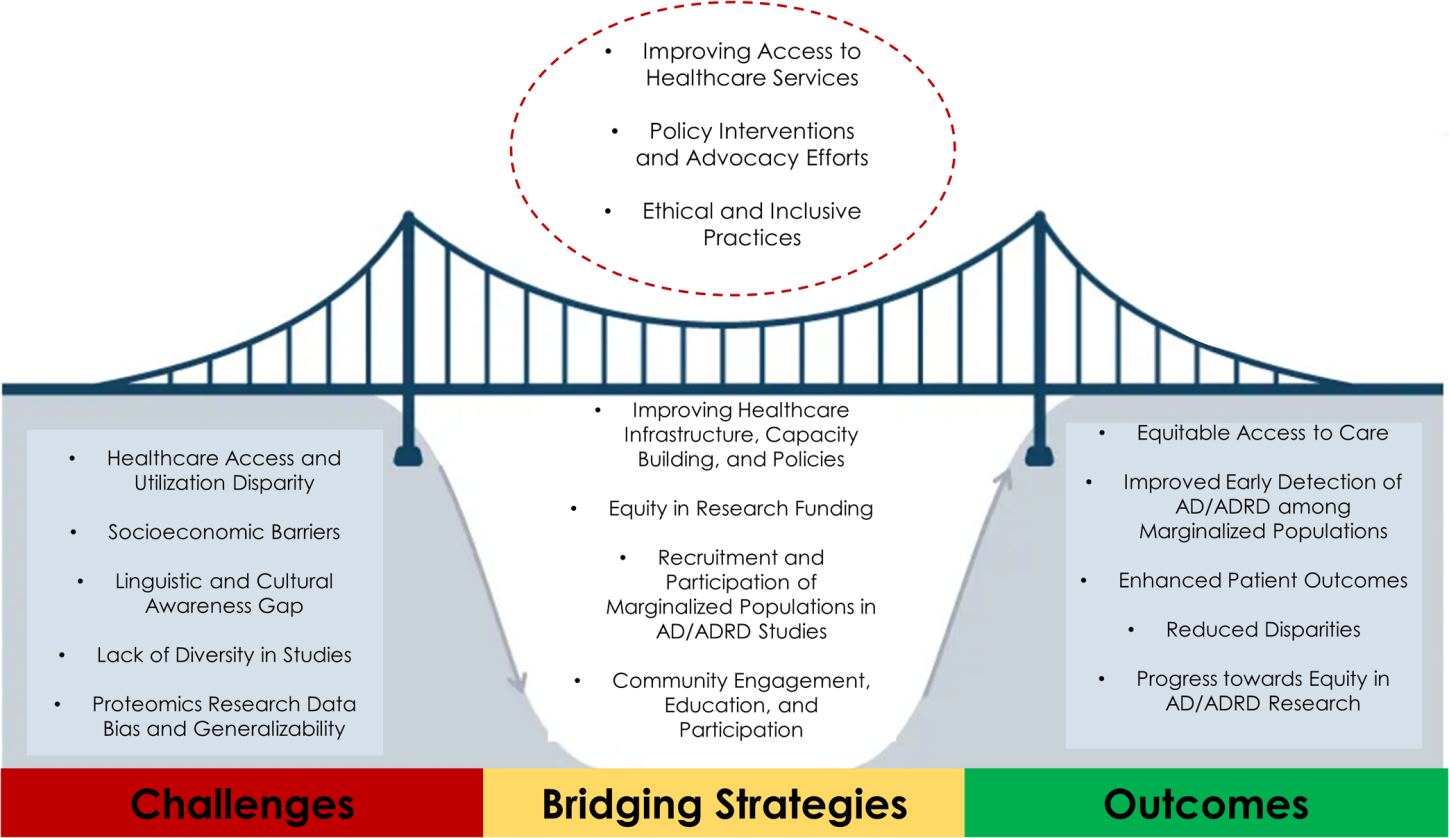
Illustration of innovative strategies to bridge health disparities for AD/ADRD among marginalized communities.

### 5.1 Improving Healthcare Infrastructure, Capacity Building, and Policies

Investments in research infrastructure, technology transfer, and training programs are essential for building scientific capacity in minority communities.^136^ This effort should include providing access to state-of-the-art proteomic technologies, establishing specimen biobanks, and deliberately supporting career development for underrepresented researchers and populations. High-quality, culturally competent care is lacking for minority populations, and thus, training providers to recognize cultural differences in how these groups of patients will perceive and report their symptoms is a strategy.^137,138^ In addition, the care delivery environment, as well as services and government policies can have significant impact on healthcare costs and who will be responsible for those costs.^139^ Due to the restrictive care access that elderly dementia patients without adequate insurance suffer, there is a need to create alternative payment models for healthcare services that are received by minoritized populations living with dementia.^140^ Taking into account the role that CP research can play in the identification and elimination of inequities in healthcare outcomes of elderly people who have dementia,^141^ new studies should develop infrastructure and inclusive design that encourages respective community diversity.^142^ Government policies, funding initiatives, and legislation should be geared toward helping to reduce (or eliminate) systemic barriers to equitable brain health. A winning strategy could leverage novel technological and data science advances, such as remote technology, monitoring devices, artificial intelligence (AI)-based tools, and smartphones, to improve healthcare access of vulnerable minority individuals living with dementia.^143–147^

### 5.2 Equity in Research Funding

The *status quo* for most US-based research reveals that participants in well-funded studies are mostly affluent, cisgender, non-Hispanic, White adults who are geographically in proximity to academic medical facilities.^148^ Governments, philanthropic organizations, and research agencies should prioritize funding for CP that solely addresses health disparities in AD/ADRD and includes diverse populations. Federal funding opportunities have catalyzed AD/ADRD disparities studies, among marginalized populations; however, there is still more work required in this space.^149^ Funding mechanisms for AD/ADRD CP should strategically attract new investigators to AD/ADRD research as currently practiced^149,150^ and incentivize collaboration with researchers from LMICs and minority-serving institutions.^151^ High-income, research-rich countries benefit from a competitive advantage in innovation and discoveries using cutting-edge proteomics technology for AD/ADRD research; however, such innovations have been severely limited by poor access to research funding in LMICs.^152^

### 5.3 Recruitment and Participation of Marginalized Populations in AD/ADRD Studies

Various innovative efforts have been proposed to accelerate the funding and enrollment of underrepresented groups in AD/ADRD research. Pena-Garcia *et al.* evaluated the recruitment of a community advisory board to improve recruitment of minoritized populations and to increase their participation in AD neuroimaging research.^153^ Their study indicated that participants enrolled via the community advisory board approach were more willing to obtain MRI and PET scans. Participation of African American/Black participants in brain health registry, proteomics research, and biobanking development has been improved by various innovative educational and digital interventions.^122,154–156^ Most of these studies are made possible only by research funding allocated to the investigators to evaluate pathways to the increase participation of minority populations in AD/ADRD research. Gilmore-Bykovskyi *et al.* investigated published reports that documented the recruitment and retention strategy for ethnic/racial minorities in AD/ADRD research.^157^ Few studies used a multifaceted approach that included community outreach efforts and demonstrated improvements in representation and diversity in their ADRD cohorts, albeit evidence of best practices for recruitment and retention of minoritized populations in AD/ADRD research was found to be low. This indicates that more prospective and longitudinal research focused on addressing disparities in AD/ADRD research participation among minority populations ought to be urgently funded by governments, philanthropic agencies, and nongovernmental organizations.

### 5.4 Community Engagement, Education, and Participation

Prompt identification and recruitment of a large cohort of healthy individuals at high risk of developing AD/ADRD is a critical challenge for AD/ADRD primary prevention trials.^158^ Thus, engaging with minority communities through culturally tailored outreach programs, community-based participatory research, asset-based community development approaches, and health literacy initiatives can enhance awareness, trust, and participation in proteomic studies.^122,155,159–162^ Involving community members in study design, recruitment, and dissemination of findings fosters ownership, control, and relevance of research efforts.^159,163^ To achieve enrichment of proteomics studies geared toward prevention of AD/ADRD among racial minorities, proactive and creative community-engagement approaches are needed.^154,164–166^ These active AD/ADRD outreach strategies should be deliberately aimed at building reciprocal community relationships that benefit both the research participants and the investigators.^167,168^ For example, using text- and video-based materials that tell AD/ADRD research participation stories of African Americans can cultivate a culturally salient strategy for participant enrollment.^169^ Established ties with community representatives and dissemination of educational materials in the communities via health fairs, word-of-mouth referrals, presentations, and flyer distribution^158^ can increase enrollment of older adults from ethnic minority groups as observed at the University of California, Davis Alzheimer’s Disease Center.^170^ It would be helpful for CP researchers to become engaged with communities or stakeholders to share their proposed research questions, study designs, and outcomes in order to receive feedback to inform study design and the perceived impact of the work.

### 5.5 Improving Access to Healthcare Services and Technologies

Novel proteomics-based diagnostic tests should be widely accessed and used by all elderly individuals, irrespective of their geographic location. This will foster early detection, risk stratification, and treatment monitoring. U.S. Alzheimer’s Association Technology Professional Interest Area cataloged four ongoing and upcoming domains of technology developments in the AD/ADRD field.^171^ These were technologies around management and caregiving; diagnostic assessment and monitoring; leisure and activity; and maintenance of function. Along with proteomics, emerging smart technologies and AI tools such as smart home systems (Google Home Hub and Amazon Alexa), driverless cars, virtual reality, and wearables will potentially drive new technology for the management of AD/ADRD.^171^ Assistive remote devices were well-accepted for the support and management of AD/ADRD^172^ and may support people living with dementia and their caregivers.

### 5.6 Ethical Considerations and Inclusive Practices

AD/ADRD researchers are expected to adhere to ethical principles of privacy protection, informed consent, and respect for cultural values when conducting proteomic studies and basic science research studies.^94,173^ Collaborative partnerships with local stakeholders and adherence to ethical guidelines promote responsible and equitable research practices.^173,174^ The degree of cognitive impairment in clinical AD/ADRD research often raises ethical issues *vis-à-vis* the capability of the patients to provide informed consent for their participation. However, evidence is emerging that AD/ADRD patients and their surrogates are able to decide and participate in clinical research in a manner that does not violate the patient’s values and culture.^174^ Innovative community-based approaches and strategic inclusive interventions, as well as well-reported informed consent-taking, are needed to strengthen methodological and ethical approaches to the recruitment of participants into clinical AD/ADRD proteomics research.^175–177^ Also, active and caring engagement with these communities to understand their needs, secure equitable access to research, and minimize potential risks of stigmatization is needed and helps to mitigate mistrust.^161,175^

## 6 CP Progress and Implications for AD/ADRD Health Disparities

Contemporary CP approaches sit on the methodological tripod of mass spectrometry (MS)-, aptamer-, and immunoaffinity-based approaches.^178,179^ Each of these techniques has distinct advantages and limitations, with MS-based methods currently being the most widely employed due to their multiplexing capabilities and high sensitivity.^180^ Aptamer- and immunoaffinity-based approaches are also gaining good traction for highly specific CP applications, especially when unique protein epitopes are being targeted.^181,182^ Recent advancements in technology and bioinformatics pipelines promote continuous evolution for clinical translational biomarker discovery.^183,184^ Despite the remarkable evolution of these proteomics techniques for AD/ADRD research, only a few FDA-approved blood diagnostics and drug treatments have emerged, as discussed earlier. Diagnostics provide hope but are only disease- modifying interventions and not a cure.^185–187^ The depth of clinically relevant and ethically compliant proteomic information that can be collected from human specimens has been significantly improved by recent advances in CP approaches and workflows.^94,178,179^ The fact that the pathological deposition of oligomeric amyloid-β plaques and hyperphosphorylated *tau* protein in neurofibrillary tangles in the brain may occur up to 20 years before clinical symptoms in AD/ADRD requires paradigm-shifting approaches to early detection, diagnosis, monitoring and diseases modification.^59^ The Lancet Commission on dementia, prevention, intervention and care, in its 2020 update, showed that up to 40% of AD/ADRD could be delayed or prevented by focusing on modifiable risk factors and advised countries to set up ambitious public health programs and policies for the control of AD/ADRD.^188^ Despite the entrenched role of CP in the discovery of various theragnostic tools for AD/ADRD, there is no clear-cut evidence of their efficacy in early detection and prompt therapy among ethnic and racial minority communities who suffer AD/ADRD. Hence, an integrated national public health approach that establishes collaboration between community organizations, public health, and health systems will improve health outcomes in these vulnerable populations.^189^

## 7 Potential Opportunities for Using CP to Address Disparities in AD/ADRD: A Case Focus

We previously demonstrated the importance of including African American individuals in AD/ADRD studies. Supervised classification, especially when used to evaluate the accuracy of proteomic biomarkers, was reliable for distinguishing Non-Hispanic White and Black adults with AD.^102^ Morris *et al.* discovered significant differences in CSF tau protein concentration of African American adults when compared with Non-Hispanic Whites adults. Among the 1255 adult CSF samples collected, it was found that the mean tau protein concentrations were 294 pg/mL and 443 pg/mL for Black and Non-Hispanic White adults, respectively.^128^ This is consistent with current evidence indicating that lower CSF levels of tau (an indicator of neuronal damage) have been reported in African Americans/Black patients with AD when compared to non-Hispanic Whites.^1,103^ This difference has been hypothesized to be related to higher symptoms of cognitive decline in Blacks despite a lower level of neuronal damage, suggesting that other physiological mechanisms potentially contribute to the susceptibility of Blacks to the development of AD, and this warrants further investigation.^103,190^ Furthermore, synaptic proteins such as VGF nerve growth factor inducible, Secretogranin II (SCG2) and Neuronal pentraxin II (NPTX2) have been found to be significantly lower in CSF samples collected from Black patients with AD as compared with samples from Non-Hispanic White AD patients.^103^ We identified 351 novel AD proteins in a brain proteomic study by including African American participants,^122^ highlighting the heterogeneity of disease and missed information when patient populations are not diverse. Heat shock protein β-1 (HSPB1), APP, and patient age were found to accurately predict AD (area under curve (AUC) range = 0.91–0.96) using four brain proteomic data sets that included African American brain samples.^191^ Population-based proteomic differences at baseline have crucial clinical management ramifications, albeit additional CP studies, are required that should include other ethnic minorities such as Hispanics, American Indians, and Alaska Natives. For instance, O’Bryant et al. demonstrated that disparities existed in proteomic profiles of neurodegeneration when comparing Mexican American to non-Hispanic White adults.^192^

Diversity-tailored funding initiatives can provide support for studies focused on underrepresented groups and facilitate community engagement, outreaches, and partnerships.^193,194^ The limitations identified in the generalized and imprecise application of current diagnostic and therapeutic approaches to the management of AD/ADRD in diverse populations can be potentially overcome by the opportunity that CP offers for understanding disease mechanisms, prediction/monitoring, and personalized management of diseases.^195,196^ Essentially, clinical proteomics is poised to offer a powerful lens for understanding molecular characteristics of AD/ADRD in specific underserved populations, thereby providing an opportunity for early detection, accurate diagnosis, tailored treatments, and a more equitable healthcare.

## 8 Conclusions and Future Directions

Health disparities in CP require urgent attention and remediation. To reduce or eliminate the disparity gaps, there is a need for international cooperation among stakeholders, such as policymakers and the general scientific communities. In addition, concerted efforts in knowledge-sharing are needed to promote the equitable use of CP to improve outcomes of AD/ADRD among vulnerable populations. Some strategies that may be employed to address AD/ADRD disparities include equitable resource allocation, research partnerships, and capacity development. Innovative alternative local and inclusive funding strategies can be leveraged to enhance the development and use of CP. For example, industrial partnership and investment can be fostered with private stakeholders such as biotechnology, instruments, and pharmaceutical companies for the implementation of biomarkers and drug target discovery. Nonprofits, charities, academic institutions, and philanthropic organizations can also be involved in CP research funding.^197,198^ This can be exemplified by the promotion of proteomics research by the Human Proteome Organization (HUPO) through international partnerships and initiatives.^199^ Local nongovernmental opportunities that can be leveraged include the integration of high-throughput and automated proteomics technologies into the diagnostic workflows of hospitals and clinical laboratories. Advocacy, community engagement, and health provider training also provide the requisite skills, acceptance, and knowledge needed to understand and utilize CP tools effectively. These alternative approaches can result in a more sustainable and diverse ecosystem. Although CP holds great promise for improving the clinical management of AD/ADRD, disparities in access to funding, technology, and research partnerships limit progress in this area of science. We can ensure that the whole world benefits from the use of CP for the management of AD/ADRD among minoritized populations only by working together.
